# Potential of rosmarinic acid to ameliorate toxic effects of diethyl methoxy thio‑phosphoryl thio‑succinate on albino wistar rats’ lung, mast cell infiltration inhibitory pathway

**DOI:** 10.1002/fsn3.2316

**Published:** 2021-06-01

**Authors:** Ahmed S. Ahmed, Marwa M. Mona, Mona A. Abdel‑Kareem, Rasha A. Elsisy

**Affiliations:** ^1^ Anatomy and Embryology Department College of Medicine Tanta University Tanta Egypt; ^2^ Medical Biochemistry and Molecular Biology Department College of Medicine Kafrelsheikh University Kafrelsheikh Egypt; ^3^ Anatomy and Embryology Department College of Medicine Kafrelsheikh University Kafrelsheikh Egypt

**Keywords:** lung, malathion, mast cell, rosmarinic acid, SP‐D gene

## Abstract

Malathion (MA) is a widely used pesticide in agriculture. It can cause toxicity in different organs of the body. Rosmarinic acid (RO) is found in rosemary extract that can be absorbed through gastrointestinal tract mucosa with potent antioxidant, and anti‐inflammatory potential. The current study is designed to investigate the potential of RO to protect the lung after MA administration. Forty albino rats were allocated equally to four groups. C‐group received corn oil. RO‐group received RO orally. MA‐group received MA. MA‐RO‐group received RO in addition to MA. After three weeks the lungs were dissected for histopathological and biochemical investigations. MA‐group showed manifestations of severe inflammation with inflammatory cells infiltration in the lung. MA‐RO‐group showed limited inflammatory cell infiltration. C‐group and RO‐group appeared with weak anti‐survivin immunoreactivity. MA‐group showed strong positive immunoreactivity. The reactivity was weakly positive in MA‐RO‐group. MA‐group showed a significant decrease in SP‐D gene expression in comparison to the C‐group, in addition, MA‐RO‐group showed a significant increase in SP‐D expression. In conclusion, the current study approves that oral administration of MA causes lung injury as it has inflammatory effects, caused by oxidative stress and reports the potential of RO to protect lung tissue against toxic effects of MA through its anti‐inflammatory, antioxidant, and anti‐apoptotic potential.

## Graphical abstract

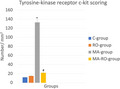

## 1 INTRODUCTION

Malathion (MA) (diethyl methoxy thio‑phosphoryl thio‑succinate) is a widely used insecticide that could be absorbed through mucous membranes or skin then converted into malaoxon (Dorri et al., 2015; Sarabia et al., 2009; Wankhade, 2012). Insecticide could cause acetylcholine accumulation (Al‐Attar, 2010) or activate the production of reactive oxygen species (ROS) (Govindarajan et al., 2019; Noaishi et al., 2013; Ozkan et al., 2014).

Rosemary is widely used in herbal medicine, that has potent anti‐inflammatory potential (Takaki et al., 2008). Rosmarinic acid (RO) is the main polyphenols found in rosemary extract (Moore et al., 2016). RO is absorbed in the gastrointestinal tract (Debersac et al., 2001; Ward, 2010). A disruption of oxidative balance was found to be important in the pathogenesis of lung inflammatory diseases, such as acute lung injury and acute respiratory distress syndrome (Crimi et al., 2006). Survivin, a member of the inhibitor of apoptosis family, inhibits caspase‐mediated cell death by increasing inhibition of caspase through binding the X‐linked inhibitor of apoptosis. (Terasaki et al., 2013). They found clear evidence of survivin‐positive epithelial cells of bronchioles and alveoli in bleomycin‐injured lungs and suggested that it may be involved in lung regeneration and proliferation after acute lung injury. Because of the excellent bioactivity of Ro, authors hypothesized that supplementation with RO may protect against MA‐induced lung injury in rats. Therefore, the current study is designed to investigate the potential of RO to protect the lung after MA administration.

## 2 MATERIALS AND METHODS

### 2.1 Kits and chemicals

All chemicals and kits were purchased from Sino‐pharm (China).

### 2.2 Animals and experimental design

Forty albino Wistar rats were used with an average of weight 200 gm. Animals were allowed to acclimatize for one week under the following conditions (in accordance with national and institutional guidelines): free access to chow and water, 12 light/dark cycles, temperature 25°C, humidity 55%. Chow and water consumption in addition to mortality and health status were recorded daily. At the end of the first week, rats were allocated into four groups (*n* = 10). Control group (C‐group) received 0.5 ml of corn oil/day by oral gavage. RO treated group (RO‐group), received RO (50 mg kg^−1^ b.w. day^−1^) (Domitrović et al., 2013) in corn oil vehicle orally by oral gavage. MA treated group (MA‐group) received MA (100 mg kg^−1^ b.w. day^−1^) in corn oil vehicle (Kalender et al., 2010). MA +RO treated group (MA‐RO‐group), received RO (50 mg kg^−1^ b.w. day^−1^) in addition to MA (100 mg kg^−1^ b.w. day^−1^) in corn oil vehicle. At the end of the treatment protocol (three weeks) all rats were euthanized by the help of sodium pentobarbital (intraperitoneal injection, 60 mg/kg b.w.). Lungs were dissected. The right lung was fixed in 10% formalin for histopathological examinations, while the left one was rapidly frozen (−80°C) for further biochemical studies.

### 2.3 Histopathological examination

Hematoxylin and eosin staining was done in accordance with Li et al., (2018). Briefly, the fresh lung was cut into 0.5 cm^3^ cubes immediately after extraction from the rats. It was placed in fixative 10% formalin and left for 48 hr then placed in tissue processing cassettes. By the help of ascending grades of alcohol, tissue is dehydrated to remove water and formalin traces from tissue then immersed in xylene to remove alcohol and facilitate paraffin wax infiltration into the tissue. Cassettes were placed on warm plates then tissue was removed and immersed in paraffin blocks. After paraffin solidification, the blocks were cut into 5 μm thick sections by using a manually operated rotary microtome. Tissue sections were placed on glass microscope slides, rehydrated, stained with hematoxylin and eosin. The stained tissue sections were dehydrated again by ascending grades of alcohol for 10 min then covered by a coverslip. Scoring was done as per lesion severity as shown in Table 1.

**TABLE 1:** Histopathological scoring criteria

Lesion	Criteria	Score
Vascular lesion	Normal blood vessels	0
	Congestion	1
	Congestion, edema and hemorrhage	2
	Loss of tunica media of blood vessels and hemorrhage	3
Pneumonia	Normal lung	0
	Mild interstitial pneumonia	1
	Moderate interstitial pneumonia	2
	Marked interstitial pneumonia	3
Alveolar patency	Normal patent alveoli	0
	Mild thickening of alveolar septa with patent alveoli	1
	Moderate thickening of septa with decreasing alveolar space	2
	Marked thickening of septa with marked obliteration of alveoli	3

### 2.4 Immunohistochemistry examinations

Immunohistochemistry was done in accordance with Magaki et al., (2019). Briefly, paraffin embedded tissue sections were sliced (5 μm thick) and mounted to charged slides. Sections were deparaffinized and rehydrated. 200 μl of diluted Primary antibody [polyclonal anti‐survivin antibody (1:400), anti‐tyrosine‐kinase receptor c‐kit antibody (1:50) – mast cell marker] were mounted to the tissue after dilution with antibody diluent as per manufacturer protocol (signal stain diluent). In next morning, slides were washed by wash buffer for 3 min then covered with two drops of Signal Stain Boost Detection Reagent followed by incubation at room temperature in a humidified chamber for 30 min. 200 μl of SignalStain^®^ DAB (Biocompare) were applied to each section. After staining, slides were immersed in distilled water then counterstained with hematoxylin to stain nuclei in blue for better visualization. Ten fields per section were analyzed by Image J 1.24 version software.

### 2.5 Real‐time quantitative polymerase chain reaction (PCR) analysis

PCR (Thermo Fisher, USA) was used for the quantification of pulmonary surfactant protein D (SP‐D) genes. Lung samples were lysed by the help of SE‐Quoia Kit (Bio‐Rad, USA) (Jonsson et al., 2009), then transcription of RNA to cDNA was performed. PCR conditions were (initial denaturation at 96°C [4 min], then forty cycles of 96°C [20 s], 63°C [30 s], and 72°C [30 s]). The primers sequences were summarized in (Table 2).

**TABLE 2:** List of primers sequences used

Gene	Forward primer sequence	Reverse primer sequence
SP‐D	ACTCATCACAGCCCACAACA	TCAGAACTCACAGATAACAAG
β ‐actin	AAGTCCCTCACCCTCCCAAAAG	AAGCAATGCTGTCACCTTCCC

### 2.6 Statistical analysis

Statistical Package for Social Sciences (SPSS) software version 20 (SPSS Inc., USA) was used for data analysis. The statistical significance of differences between groups was validated using one‐way analysis of variance (ANOVA) (Lee & Lee, 2018). Post hoc Tukey–Kramer test was used for group comparison (Kim, 2015). Data were expressed in mean ±standard deviation and probability value was considered significant if <0.05.

## 3 RESULTS

### 3.1 Effect of RO on lung histological architecture after MA administration

**FIGURE 1:**
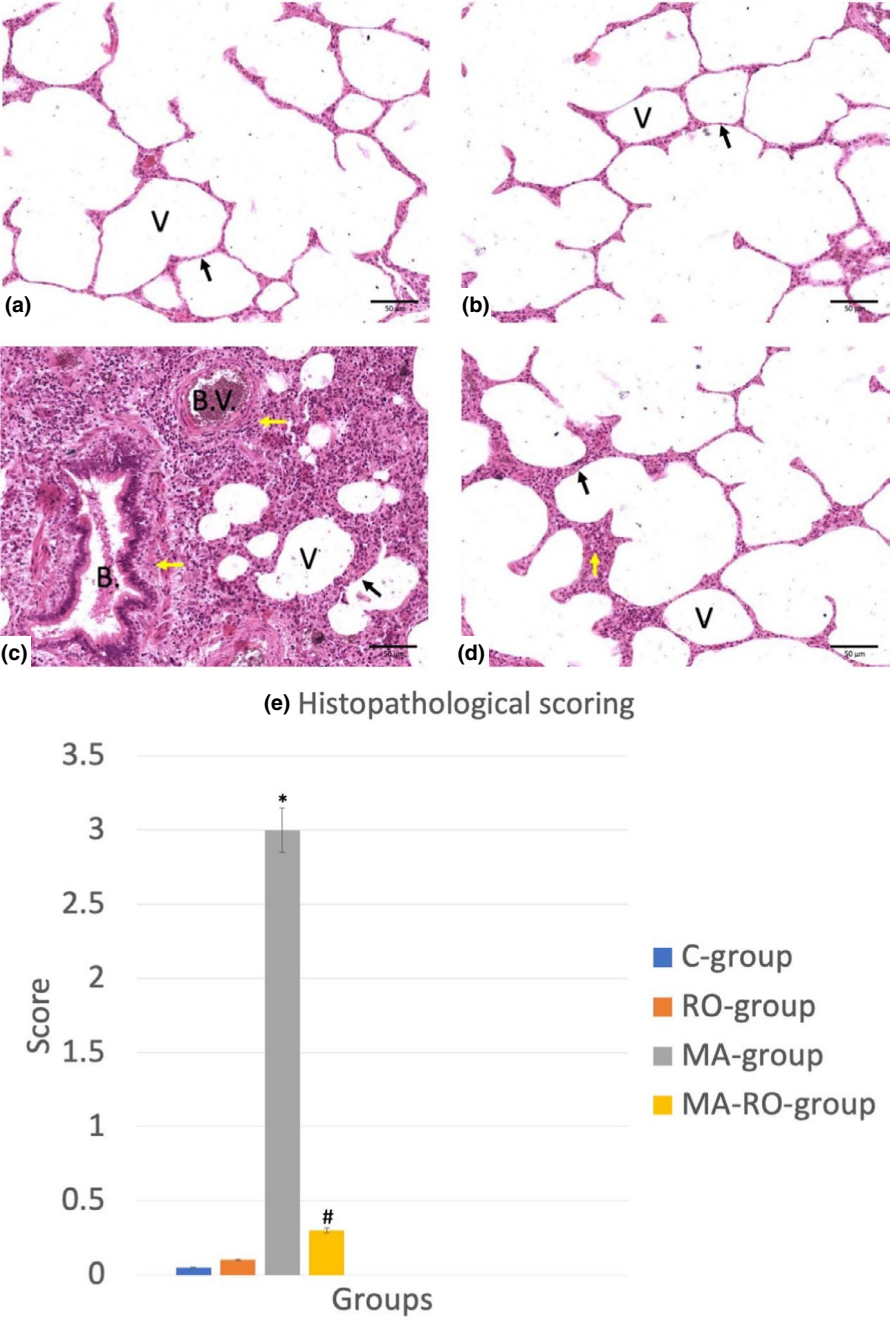
(a–d) Photomicrographs of lung stained with hematoxylin and eosin (X 400), (*n* = 10). (a, b) Represents C‐group and RO‐group respectively with normal histological architecture. (c) Represents MA‐group with manifestations of severe inflammation with inflammatory cells infiltration in addition to areas of hemorrhage. The width of most alveoli appears decreased, few alveoli are completely obliterated. (d) Represents MA‐RO‐group with regain of the normal histological architecture. (Note: V = alveoli, B. = bronchioles, B.V. = blood vessels, Black arrow =interalveolar septa, Yellow arrow=inflammatory cells infiltration). (E) Represents histopathological scoring with significant (*p* <.05) increase in MA‐group if compared to C‐group while MA‐RO‐group showed a significant (*p* <.05) decrease if compared to MA‐group. * significant (*p* <.05) difference in comparison to C‐group. # significant (*p* <.05) difference in comparison to MA‐group. Data are presented as mean ± *SD*, (*n* = 10)

Lungs of both C‐group and RO‐group had normal architecture with intact patent alveoli with spongy appearance in addition to normal blood vessels and bronchioles. MA‐group showed manifestations of severe inflammation with inflammatory cells infiltration (in the interalveolar interstitial tissues, peri‐bronchial spaces, and peri‐vascular spaces) in addition to areas of hemorrhage. The width of most alveoli was decreased, few alveoli were completely obliterated. MA‐RO‐group showed regain of the normal histological architecture with limited inflammatory cells infiltration. Histopathological scoring showed a significant increase in MA‐group if compared with C‐group while MA‐RO‐group showed a significant decrease in comparison to MA‐group (Figure 1).

### 3.2 Effect of RO on mast cells infiltration after MA administration

**FIGURE 2:**
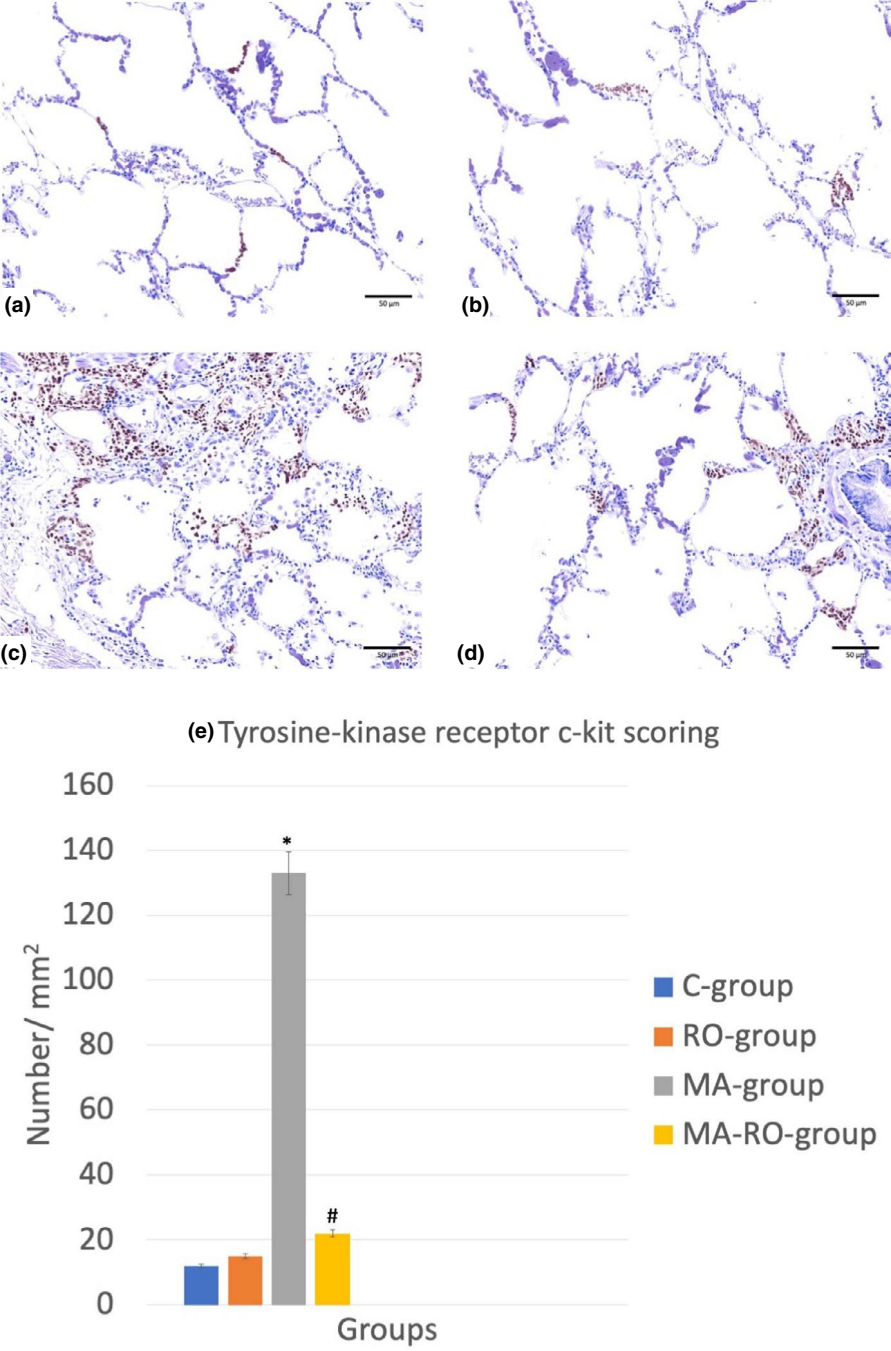
(a–d) Photomicrographs of lung stained with anti‐tyrosine‐kinase receptor c‐kit antibody (X 400), (*n* = 10). C‐group (a) and RO‐group (b) appear with weak immunoreactivity. MA‐group (c) shows strong positive immunoreactivity. The reactivity is weak positive in MA‐RO‐group (d). (e) Scoring shows a significant (*p* <.05) increase in MA‐group if compared to C‐group while MA‐RO‐group shows a significant (*p* <.05) decrease if compared to MA‐group. * significant (*p* <.05) difference in comparison to C‐group. # significant (*p* <.05) difference in comparison to MA‐group. Data are presented as mean ± *SD*, (*n* = 10)

Histopathological examination of tissue sections stained with anti‐tyrosine‐kinase receptor c‐kit antibody showed C‐group and RO‐group appeared with weak immunoreactivity. MA‐group showed strong positive immunoreactivity denoting severe mast cells infiltration. The reactivity was weakly positive in MA‐RO‐group. Scoring showed a significant increase in MA‐group if compared to C‐group while MA‐RO‐group showed a significant decrease if compared to MA‐group (Figure 2).

### 3.3 Effect of RO on survivin immunoreactivity after MA administration

**FIGURE 3:**
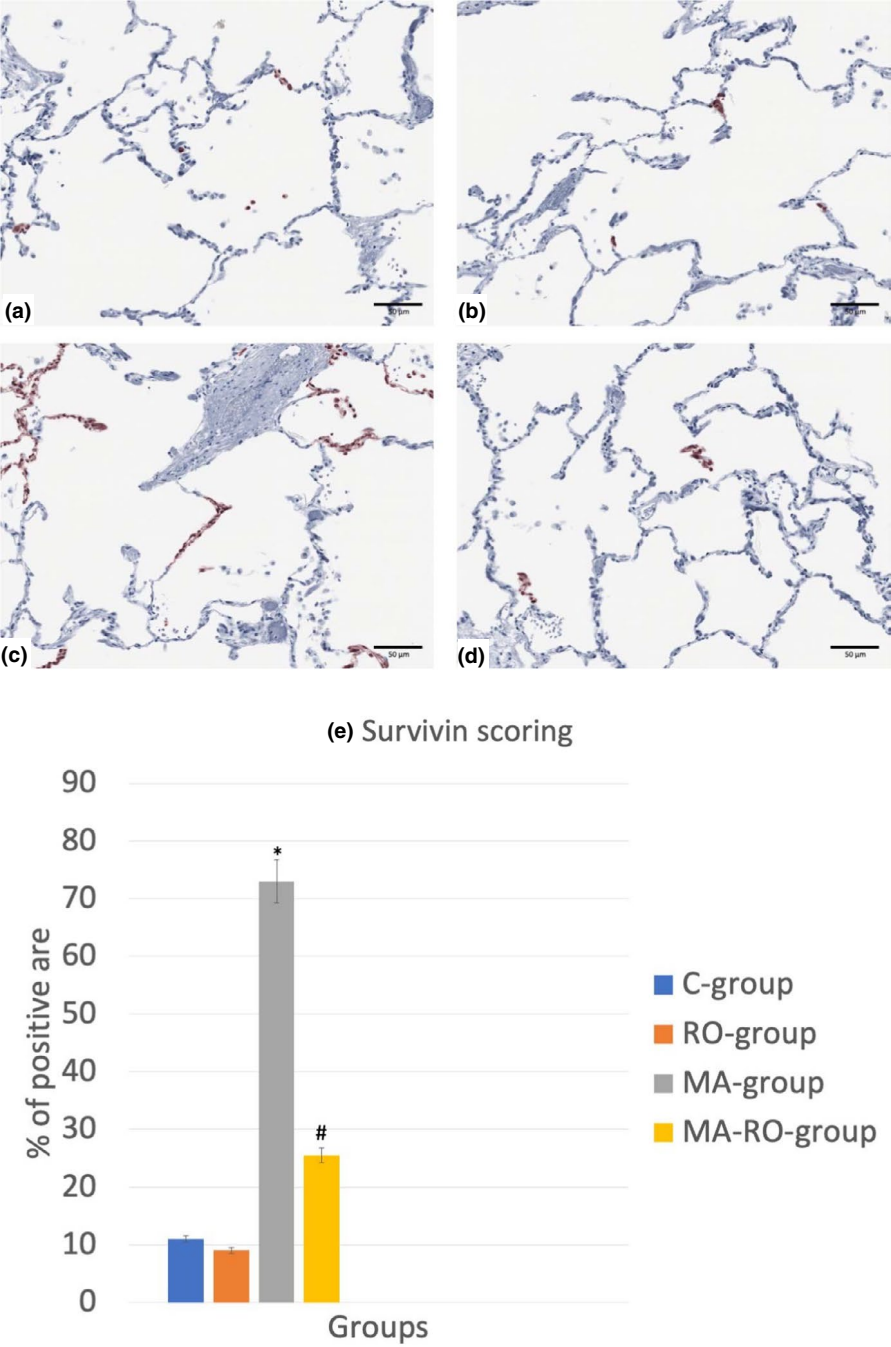
(a–d) Photomicrographs of lung stained with anti‐survivin antibody (X 400), (*n* = 10). C‐group (a) and RO‐group (b) appear with weak immunoreactivity. MA‐group (c) shows strong positive immunoreactivity. The reactivity is weak positive in MA‐RO‐group (d). (e) Scoring shows a significant (*p* <.05) increase in MA‐group if compared to C‐group while MA‐RO‐group shows a significant (*p* <.05) decrease if compared to MA‐group. * significant (*p* <.05) difference in comparison to C‐group. # significant (*p* <.05) difference in comparison to MA‐group. Data are presented as mean ± *SD*, (*n* = 10)

Histopathological examination of tissue sections stained with anti‐survivin antibody showed C‐group and RO‐group appeared with weak immunoreactivity. MA‐group showed strong positive immunoreactivity. The reactivity was weakly positive in MA‐RO‐group. Scoring showed a significant increase in MA‐group if compared to C‐group while MA‐RO‐group showed a significant decrease if compared to MA‐group (Figure 3).

### 3.4 Effect of RO on SP‐D gene expression after MA administration

**FIGURE 4:**
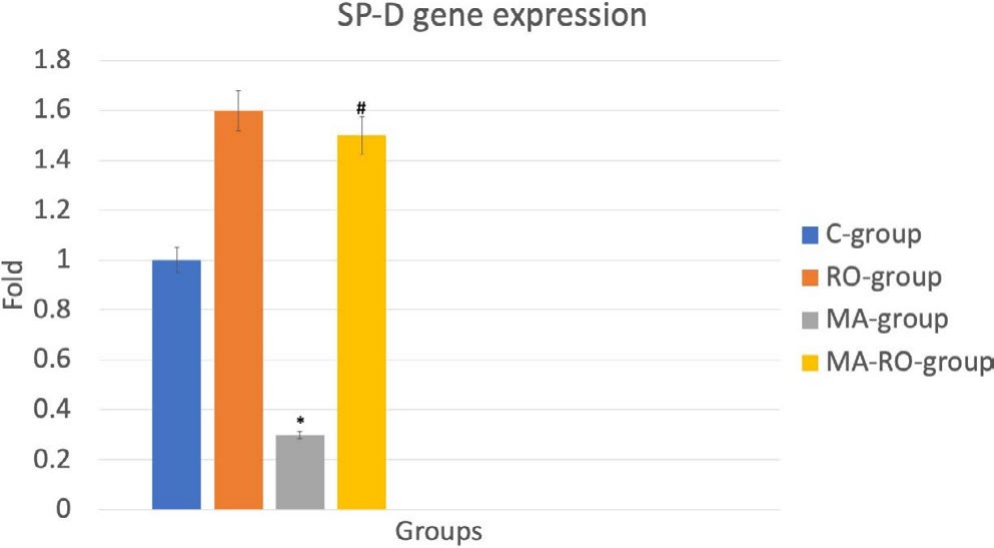
Effect of RO on SP‐D gene expression after MA administration. MA‐group shows a significant (*p* <.05) decrease of SP‐D gene expression if compared to C‐group. There is a significant increase (*p* <.05) in gene expression of MA‐RO‐group if compared to MA‐group. * significant (*p* <.05) difference in comparison to C‐group. # significant (*p* <.05) difference in comparison to MA‐group. Data are presented as mean ± *SD*, (*n* = 10)

MA‐group showed a significant decrease of SP‐D gene expression if compared to C‐group, in addition, there was a significant increase in gene expression of MA‐RO‐group if compared to MA‐group (Figure 4).

## 4 DISCUSSION

Currently, MA is an extensively used member of organophosphorus (Ops) family (Bogen & Singhal, 2017; Ozsoy et al., 2016) which can cause injury to the lung (Angelini et al., 2013; Atiş et al., 2002; Moin‐Azad Tehrani et al., 2011; Nambiar et al., 2007). Uysal and Karaman (2018) reported that MA exposure could initiate oxidative stress and apoptosis. Kim et al., (2005), Li et al., (2010) and Sui et al., (2012) documented that RO could ameliorate fibrotic, apoptotic, and oxidative pathways in the pulmonary tissue. Mammals can be adversely affected by malathion MAL through nearly any route of exposure, including the oral ingestion of food and drinking water contaminated with MAL (Akbel et al., 2018; Sapbamrer & Hongsibsong, 2014). MAL is rapidly absorbed, through different routes, and distributed to different body organs, thus leads to several pathologies (Selmi et al., 2018). Uysal and Karaman (2018) revealed that acute oral malathion administration increased oxidative stress and apoptosis in the lung tissue of rats.

In current study, histological examination of MA‐group showed manifestations of severe inflammation with inflammatory cells infiltration in the interalveolar interstitial tissues, peri‐bronchial spaces, and peri‐vascular spaces in addition to areas of hemorrhage. The width of most alveoli was decreased, few alveoli were completely obliterated. Histopathological scoring showed a significant increase in MA‐group if compared to C‐group. These results come in consistent with Toś‐Luty et al., (2003) who mentioned that the lungs of MA‐group showed infiltrations and widening of interalveolar septa with the presence of single pulmonary phagocytes. These signs of inflammation were reported by Malaviya et al., (2010) who reported the potential of sulfur mustard to induce severe inflammatory manifestations in lung. In addition, Amara et al., (2012) reported the destructive effect of dimethoate (DM) (one of organophosphate insecticide) administration on lung histological architecture in the form of alveolar hemorrhage, hemosiderin deposits, and emphysema. In current study, authors hypothesized that the changes caused by MA administration may be due to reactive oxygen species activation or inflammatory cells infiltration as explained by Amara et al., (2012) or due to oxidative stress as mentioned by Possamai et al., (2007) and Uysal and Karaman (2018).

Histological examination of MA‐RO‐group showed regain of the normal histological architecture with limited inflammatory cells infiltration. Histopathological scoring of the same group showed a significant decrease if compared to MA‐group. This indicates anti‐inflammatory effect of RO and its potential to ameliorate the oxidative stress caused by MA.

This comes into agreement with Sanbongi et al., (2003) who observed that RO inhibited pathophysiological changes such as neutrophilic inflammation and edema in the lung. In addition, Chu et al., (2012) reported the potential of RA to inhibit tumor necrosis factor‐α (TNFα). Moreover, Rocha et al., (2015) demonstrated the potential of RO to reduce the release of pro‐inflammatory cytokines.

In current study, examination of tissue sections stained with anti‐tyrosine‐kinase receptor c‐kit antibody showed strong positive immunoreactivity in MA‐group while the reactivity was weakly positive in MA‐RO‐group. Scoring showed a significant increase in MA‐group if compared to C‐group while MA‐RO‐group showed a significant decrease if compared to MA‐group. These changes denoting severe mast cells infiltration in MA‐group as anti‐tyrosine‐kinase receptor c‐kit antibody is a diagnostic marker of mast cell (Leong et al., 2003). This indicated the inflammatory effect of MA, as mast cells are important for immune responses as in allergy, asthma, and arthritis (Galli et al., 2008; Kaur et al., 2006). In addition, mast cell activation can enhance oxidative stress pathways (Zhao et al., 2014). Weak positive reactivity in MA‐RO‐group confirmed the anti‐inflammatory and antioxidant effect of RO.

In current study, histopathological examination of tissue sections stained with anti‐survivin antibody showed that MA‐group had strong positive immunoreactivity which was weakly positive in MA‐RO‐group. Scoring showed a significant increase in MA‐group if compared to C‐group while MA‐RO‐group showed a significant decrease if compared to MA‐group. This revealed the anti‐inflammatory and antioxidant activities of RO and its role in MA‐induced lung injury. These results were coincided with Ahmed et al., (2019) who found that silymarin led to the resolution of acute lung injury (ALI) with subsequent decrease in lung tissue survivin immunostaining. In addition, Terasaki et al. (2013) reported that survivin was upregulated in ALI induced by bleomycin and was considered as the key mediator of cytoprotection. Moreover, Amenomori et al., (2011) demonstrated that survivin increased after lipopolysaccharide (LPS) induced ALI in mice and its level was decreased with damage resolution.

In current results, MA‐group showed a significant decrease of SP‐D gene [located in type II pneumocytes (Kasper et al., 2002)] expression if compared to C‐group, in addition, there was a significant increase in the same gene expression in MA‐RO‐group if compared to MA‐group. Decreased expression of SP‐D gene in MA‐group revealed the apoptotic effect of MA which comes in agreement with Clark et al., (2002) who linked between SP‐D deficiency and apoptosis of pneumocytes, so significant increase in gene expression of MA‐RO‐group proved that RO has anti‐apoptotic effect on MA‐induced lung injury. To the best of our knowledge, this is the first study to report the anti‐apoptotic potential of RO against MA on lung tissue. Other studies had shown the anti‐apoptotic effect on the cardiac muscles (Kim et al., 2005), and myoblast C2C12 cell line (Chen et al., 2014). Another study showed that RO arrested apoptosis induced by a high‐fat diet (Cai et al., 2019). On the other hand, many studies demonstrated the apoptotic effect of RO on cancer cells such as lung, and prostate (Yesil‐Celiktas et al., 2010).

In conclusion, the current study approves that oral administration of MA causes lung injury as it has inflammatory effects, caused by oxidative stress and reports the potential of RO to protect lung tissue against toxic effects of MA through its anti‐inflammatory, antioxidant, and anti‐apoptotic potential.

## CONFLICT OF INTEREST

Authors have declared that no competing interests exist.

## AUTHOR CONTRIBUTIONS

**Ahmed A. Ahmed:** Conceptualization (equal); Data curation (equal); Formal analysis (equal); Investigation (equal); Methodology (equal); Project administration (equal); Resources (equal); Software (equal); Supervision (equal); Validation (equal); Visualization (equal); Writing‐original draft (equal); Writing‐review & editing (equal). **Marwa** **M. Mona** **:** Data curation (equal); Methodology (equal); Writing‐review & editing (equal). **Mona A. Abdel‑Kareem:** Formal analysis (equal); Investigation (equal); Methodology (equal); Validation (equal); Visualization (equal); Writing‐original draft (equal). **Rasha A. Elsisy:** Conceptualization (equal); Formal analysis (equal); Investigation (equal); Supervision (equal); Visualization (equal); Writing‐review & editing (equal).

## ETHICS STATEMENT

The study protocol was approved by Research and Ethics Committee, Quality Assurance Unit, Faculty of Medicine, Tanta University, Egypt.

## Data Availability

The datasets generated during and analyzed during the current study are available from the corresponding author on reasonable request.
